# The Effects of Selected Mechanical and Anthropometric Variables on Change-of-Direction Ability in National Team-Level Youth Basketball Players

**DOI:** 10.3390/sports14040129

**Published:** 2026-03-25

**Authors:** Áron Mészáros, Bence Kopper, Annamária Zsákai, József Horváth, Lukasz Trzaskoma, Tamás Szabó

**Affiliations:** 1 Doctoral School of Sport Sciences, Hungarian University of Sport Science, 1123 Budapest, Hungary; 2 Hungarian Basketball Federation, 2040 Budaörs, Hungary; lukasztrzaskoma77@gmail.com; 3 Department of Kineziology, Hungarian University of Sport Science, 1123 Budapest, Hungary; 4 Hungarian Handball Federation, Sport Sciences and Diagnostic Research Centre, 1103 Budapest, Hungaryszabo.tamas@mksz.hu (T.S.); 5 Department of Biological Anthropology, Eötvös Loránd University, ELTE, 1117 Budapest, Hungary; 6 Faculty of Physical Education, Department of Theory of Sport, Józef Pilsudski University of Physical Education in Warsaw, 00-968 Warsaw, Poland

**Keywords:** change of direction, deceleration, centripetal force, angular speed, acceleration, countermovement jump, youth basketball

## Abstract

Change-of-direction (COD) ability is a key determinant of performance in youth basketball, yet the relative contribution of braking, re-acceleration, trunk motion, and body composition remains unclear. Thirty-two male U18 national-team level players (17.6 ± 0.7 y; 194.8 ± 4.5 cm; 89.1 ± 9.4 kg) completed whole-body and segmental DEXA assessment, bilateral countermovement jump (CMJ) testing and a 505 agility test (505) instrumented with a local positioning system. Mean COD times were 2.36 ± 0.09 s (505) and 1.84 ± 0.08 s (303), with maximal deceleration (DcMax) of −7.26 ± 0.52 m·s^−2^. Paired *t*-tests showed no significant differences between right- and left-leg turns for any variable (all *p* > 0.25), indicating symmetrical COD performance. General linear models revealed that DcMax was the only consistent predictor of COD time (505: R^2^ = 0.53, F (7,24) = 3.91, *p* = 0.006, partial η^2^ = 0.31; 303: R^2^ = 0.49, F(9,22) = 2.34, *p* = 0.050, partial η^2^ = 0.34), with a smaller additional effect of approach speed for the 303 segment (*p* = 0.049). Body-composition indices and CMJ variables showed only weak, non-significant correlations with COD time (|r| < 0.30, *p* > 0.05), and neither centripetal force nor trunk angular speed was associated with performance. These findings indicate that high-intensity braking capacity, rather than muscle mass or jump power per se, is the primary mechanical determinant of COD in elite youth basketball, suggesting that deceleration-focused training should be prioritized in performance development.

## 1. Introduction

The ability to rapidly change direction (COD) is a fundamental component of performance in contact team sports such as basketball, handball, and football. Previous research has examined COD from multiple perspectives, including cognitive decision-making processes [1,2,3,4], neuromuscular strength, eccentric braking capacity, reactive strength, movement technique, and the influence of anthropometric and body-composition characteristics. These factors collectively contribute to the mechanical and perceptual demands of COD actions in basketball. In our research, the focus was on physical abilities and anthropometric characteristics. In 2000, FIBA (International Basketball Federation) reduced the offence time in basketball to 24 s, following the regulation in place in the NBA since 1954. With this change and the continuous development of the players’ physical performance, a clear trend can be observed, whereby short dynamic actions such as acceleration, deceleration, and change of direction characterize these sports instead of straight-line sprinting ability in most real game situations. This trend was confirmed by Aldebkrim in his research conducted after the changes [5], where an action involving a change of direction or change of speed occurred every 2 s (1051 actions/game on average), regardless of the fact that players spent only 8.8% of their playing time performing high-intensity movements, like sprinting and jumping. Over recent decades, team sport matches have become even faster. In game situations, a player currently performs on average 20–30 high-intensity accelerations and decelerations, with the exact number being strongly influenced by playing position [6]. Accelerations occur more frequently than decelerations; however, decelerations are executed at higher intensity [6]. In offence, a well-timed and technically well-executed COD can create an advantageous situation on the court, but this movement element is also dominant on the defensive side. Consequently, the importance of teaching COD has increased, and coaches must pay heightened attention to developing this aspect of agility [7].

Some studies and practical experience suggest that improvements in straight-line sprint speed and acceleration can primarily be achieved through shorter ground contact times and the application of greater vertical and horizontal forces to the ground [8]. However, several studies indicate that sprinting ability itself has a weak influence on COD performance [9,10,11]. Young [10] and Webb, Lander, and Young [12,13] generally found weak correlations between maximal strength and COD time. Previous findings suggest that maximal strength does not always translate into superior COD performance, particularly in tests that require rapid deceleration and re-acceleration over short distances. In such contexts, eccentric strength and braking impulse appear to be more influential determinants than maximal concentric force production. Studies have shown that athletes with high maximal strength may not demonstrate correspondingly fast COD times if their braking technique, eccentric capacity, or ability to apply force effectively during deceleration is limited. This supports the view that sprinting ability or maximal strength alone are insufficient predictors of COD performance, especially in tasks involving sharp angles or high braking demands. In addition, core strength has been identified as an important contributor to stability and force transfer during directional changes [14,15].

The use of agility-type tests involving COD has become widespread over recent decades. In basketball, summaries of previous research indicate that such measurements are important markers for talent identification [16]. However, in order to obtain further relevant information regarding COD, new testing protocols need to be defined. Short-duration movements have become increasingly intense in basketball as well, and game situations require rapid actions, making the role of COD decisive. Earlier testing protocols used to assess COD included a substantial amount of running, which creates an evaluation problem when the specific aim is to focus on COD performance [17]. Commonly used agility tests are complex tests involving longer running distances and sometimes multiple turns; however, the more complex a test is, the more complex its outcome becomes.

With the research techniques previously available and commonly used, specialists were typically able to examine the relationship between sprinting ability and COD time, attempting to derive conclusions from this, or they obtained new information through motion analysis. These measurement methodologies did not yield results regarding deceleration, acceleration, the role of the trunk or optimal turning technique. In modern sport, increasingly advanced technological possibilities are available for monitoring players, and the use of GPS-based systems has become indispensable for identifying new training methods [18]. In indoor ball sports such as basketball, similar systems with direct signal transmission are used. These systems enable the measurement of numerous new variables, even in game situations, thereby helping us to better understand important movement elements such as COD.

The primary aim of this study was to examine the associations between change-of-direction (COD) performance and a set of biomechanical and physical performance variables. COD performance was operationalized as the time recorded in the 505 test (primary outcome) and the 303 test (secondary outcome). The explanatory variables included LPS-derived metrics (e.g., deceleration, trunk rotation speed, and CPF), sprint performance, and countermovement jump variables.

A secondary aim was to determine whether LPS-derived variables provide additional explanatory value for COD performance beyond traditional field-based measures, such as sprint times and CMJ performance.

## 2. Materials and Methods

### 2.1. Participants

A total of 32 Hungarian male basketball players participated in the research, all of them U18 players from elite Hungarian basketball academies. All players have been playing basketball for at least 6 years and are familiar with basketball movements (Table 1). All athletes competed at the highest national youth level. Although positional roles in modern basketball have become increasingly fluid, the nominal playing-position distribution was as follows: 16 guards, 11 forwards and 5 centres. Testing was conducted during the in-season period. Inclusion criteria were: (i) full participation in regular team training, (ii) no lower-limb injury in the preceding six weeks, and (iii) no current musculoskeletal complaints. Players who were injured or unable to complete maximal-effort testing were excluded. Biological maturation status was not assessed, which is acknowledged as a limitation.

The measurements for the research were performed under the supervision of the Hungarian Basketball Federation; all participants received basic information about the purpose of the study, and written informed consent was obtained from them or their legal guardians. The examinations were carried out following the rules of the Declaration of Helsinki. None of the participants could potentially be identified. The research project was supported by the Hungarian Basketball Federation and the Hungarian University of Physical Education and Sport Sciences.

### 2.2. Anthropometry and Body Composition

Data on somatic characteristics and body composition were recorded at the Sports Science and Diagnostic Research Centre of the Hungarian Handball Federation (Budapest), using a GE Prodigy DEXA device (GE Healthcare, Madison, WI, USA). Body composition was assessed using a GE Lunar Prodigy DEXA scanner (GE Healthcare, Madison, WI, USA). The device was calibrated each morning prior to testing using the manufacturer-supplied anthropometric phantom, following GE’s standard quality-control procedures. All scans were performed by a certified DEXA operator with formal qualifications and experience in densitometry. Whole-body scans were used for all participants. Standardized pre-testing conditions were applied: participants refrained from fluid intake for at least 1 h before scanning, removed all metal objects, and were positioned according to GE’s recommended whole-body protocol, with arms placed alongside the body and feet secured to minimize movement.

### 2.3. Performance Tests

Two tasks were defined to assess functional indicators: (1) CMJ test on a force plate, (2) 505 (303) agility test. Before the motor tests, all players performed the same warm-up; the 10 min protocol mainly served to warm up the lower limbs. Before data collection, all athletes completed two familiarization trials for each test. During the testing session, three recorded trials were performed for each leg (right and left), with 30–60 s rest between attempts. The best performance from the three trials was used for analysis, and these procedures were applied consistently for the symmetry assessment.

#### 2.3.1. Countermovement Jump (CMJ)

First, we recorded the results of three maximal CMJs on a 3-dimensional measuring platform (PSJ 605 typ.) and included the best experimental result (Hmax) in the evaluation. CMJ performance was assessed using the internationally accepted standard protocol: participants performed the jumps with their hands on their hips, using a self-selected countermovement depth without a pause between the eccentric and concentric phases. Three maximal trials were completed with 30–60 s rest between attempts, and the best jump height was used for analysis. Among the jump parameters, the present study used the measurements of the variables shown in Table 2.

#### 2.3.2. 505 Agility Test (505/303)

**Figure 1:**
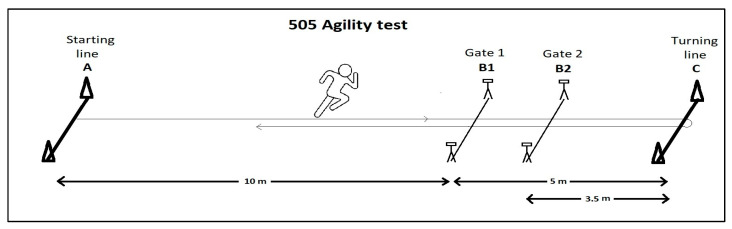
Schematic representation of the 505 and 303 change-of-direction tests used in the study.

The components of changing direction were examined using a specific motor test. A schematic representation of the test is shown in Figure 1.

The 505 agility test had to be performed at maximum speed, starting from a standing position with a 15 m sprint, followed by a 180-degree turn when one foot must catch the turning line, then a 5 m sprint in the opposite direction. Trials where the planting leg did not reach the line were not included in the evaluation. Players completed three runs for the test with each leg; in the analysis, we used the best time result among the repetitions. The times were measured with 2 pairs of light gates (Babály Timing, Budapest, Hungary) with decimal-second accuracy. The light gates were placed in the position shown in Figure 1. The time result of the distance “B1”-“C”-“B1” gave the result of the 505 test (Time 505); additionally, the time of the shorter distance “B2”-“C”-“B2” (Time 303) was also measured and used in the statistical calculations.

During the measurements of the 505 agility test, we used a WIMU PRO TM RealTrack System (Almeria, Spain) to monitor the players and collect more data. Each player wore an adjustable top with the same micro device (81 × 45 × 15 mm, 70 g), which was positioned between both scapulas on the centre of the upper back. The device has a FIFA certificate for use in official competitions and contain different sensors: (a) four triaxial accelerometers (1000 Hz) with a full-scale output range of _16, _16, _32, and _400 g; (b) three triaxial gyroscopes (1000 Hz) with a full-scale output range of 2000 degrees/seconds; (c) a three-dimensional (3D) magnetometer; (d) a 10 Hz GPS chip; and (e) a 20 Hz UWB chip. The location where the measurement was conducted was equipped with 6 calibrated antennas, which had the ability to communicate wirelessly via Bluetooth. Previous research verified the reliability of the system [19], with WIMU PRO TM showing good accuracy (for the x-position: 5.2 ± 3.1 cm and for the y-position: 5.8 ± 2.3 cm) and inter- and intra-unit reliability for ultra-wideband positioning (for the x-coordinate ICC = 0.65 and for the y-coordinate ICC = 0.85). This study, conducted on a FIBA-certified basketball court and applied movement elements that are also used in basketball, proved the reliability of the device, so we accepted it for our measurements as well.

In the case of our measurements, the accelerometer, gyroscope and magnetometer sampling frequency was 100 Hz. Of the more than 150 measured and calculated variables, we used speed, acceleration/deceleration data, as well as centripetal force and angular speed. The system provides speed and acceleration data using LPS triangulation (communication frequency is 18 Hz). Centripetal force in the WIMU system is an indicator used to support the monitoring of the COD, which is based on the force generated to the ground during turning and the acceleration rate; previous research often referred to this same indicator [20,21]. Angular speed is the speed of the trunk rotation during the turn, which the system measures using the rotational movement of the gyroscopes and determines as angular velocity.

### 2.4. Statistical Analysis

Based on the descriptive statistical results of the selected variables, we defined the following analysis tasks:

1. What is the relationship between the measured variables? Procedure used: Pearson correlation.
2. What structure do the results of 505 (303) show? Are their patterns different or similar? Procedure used: hierarchical cluster analysis. Hierarchical cluster analysis was used to generate clusters of variables using Ward’s method of measuring squared Euclidean distance by running performance, kinetics and body structural parameters. The Euclidean distance was used to estimate the variables’ relationship; for each variable, the squared Euclidean distance to the cluster means was calculated. The agglomeration schedule was used in the cluster analysis for understanding the underlying structure of the data.
3. In terms of examining the speed of execution, which variables have a major impact on the result? Procedure used: General Linear Model (GLM). Multicollinearity was assessed for all predictors included in the general linear models using the variance inflation factor (VIF) and tolerance statistics. VIF and tolerance values were extracted from the collinearity diagnostics output of the regression procedure.
4. What is the relationship between DcMax (maximum deceleration) and CMJ parameters that characterize explosive strength? Procedure used: Pearson correlation.

SPSS v. 27 was used for statistical analysis. Normality was assessed using the Shapiro–Wilk test, which is recommended for small to moderate sample sizes due to its high statistical power (the studied continuous variables had normal distribution). All variables showed normal distribution based on the Shapiro–Wilk test, and scatterplots confirmed linear relationships between the variables. Therefore, the assumptions for Pearson’s correlation were met; Pearson’s correlation was used to quantify the associations between the variables. To control multiple comparisons, *p*-values were adjusted using the Benjamini–Hochberg false discovery rate (FDR) procedure. In total, 66 pairwise correlations were tested. Adjusted *p*-values (q-values) are reported, and statistical significance was defined as q < 0.05. A paired *t*-test was used to compare the performance parameters between the right and left sides. Effect sizes were expressed as Cohen’s d (only negligible and small effects were found: 0.00–0.19—negligible effect, 0.20–0.49—small effect) in the paired *t*-test, as partial eta squared in the general linear model, and as Pearson’s correlation coefficient (r) in correlation analysis. Two separate hierarchical cluster analyses were conducted, each including nine variables. In the first analysis, the time 505 variable was included; in the second, the time 303 variable replaced it. Hierarchical clustering was performed using average linkage and squared Euclidean distance. Because SPSS does not provide internal validation indices for hierarchical clustering, the distance matrices were exported, and the cophenetic correlation coefficient and silhouette width were computed externally. The number of clusters was determined based on the dendrogram structure, the agglomeration schedule, and these validation metrics. Given the sample size (n = 32), the clustering should be interpreted as exploratory. A general linear model was used to determine which variables predicted time performance in the agility test. Although the number of predictors relative to the sample size was modest (Time 505: 7 predictors for 32 cases; Time 303: 9 predictors for 32 cases), all predictors were theoretically justified based on prior literature on COD performance. To evaluate potential instability due to model complexity, we examined multicollinearity (VIF and tolerance), adjusted R^2^, and standard regression diagnostics. Hypotheses were tested at 5% level of significance.

## 3. Results

### 3.1. Symmetry Between Right and Left Turns

The results of the functional tests in the basic statistics were broken down to compare left and right turns. Our intention was twofold: on the one hand, we wanted to examine possible asymmetry, and on the other hand, we also wanted to reduce the number of variables. The results are summarized in Table 3.

### 3.2. Bilateral Asymmetry in Performance Parameters

A paired *t*-test was used to compare the performance parameters on the right and left sides (n = 32, df = 31). Based on the results, no statistically significant difference between the two sides can be detected for any of the variables examined. The *p*-values range between 0.254 and 0.886, so all values significantly exceed the conventionally accepted significance level of α = 0.05. Accordingly, we retained the null hypothesis that the performance on the right and left sides does not differ from each other for all variables.

The 95% confidence intervals overlap zero for all pairs, supporting the non-significant difference and indicating that the direction and magnitude of the differences observed in the sample can be considered as uncertain, random fluctuations. The absolute magnitude of the t-values is also low (−1.23 < t < 1.17), reflecting the small magnitude of the effect. Based on this, it can be assumed that the differences between the two sides do not reach the practically relevant threshold.

Overall, these results suggest that the ability to change direction in the examined athletes is symmetrical, and neither the initial phase of the movement (braking phase), nor the turn requiring centripetal load, nor the subsequent acceleration phase shows any deviations indicating side preference. Hereafter, we used the average of the performed experiments with both directions.

### 3.3. Correlation Structure Between Anthropometry and COD Variables

Based on Pearson correlations, very strong and strong connections were found between body size and muscle mass indices (r = 0.533–0.956), which well reflect the structured association of anthropometric parameters. Running performance times also show a close relationship (505–303: r = 0.958), indicating that the two metrics capture the same performance component, confirming the results of the *t*-test.

Several moderate-to-strong correlations can be identified between the kinetic and kinematic variables of the turn (e.g., Sp1 and DcMax: r = −0.718; DcMax and AcMax: r = −0.636), indicating that the braking, angular velocity, and acceleration characteristics form a functional unit. These patterns indicate that the variables associated with COD performance tend to cluster into well-defined, interconnected groups. At first glance, the positive correlation between test time (Time 505 and Time 303) and braking force may appear counterintuitive, as it would suggest that greater deceleration is linked to longer execution times. However, because deceleration values are expressed as negative numbers (see Table 4), a lower numerical value actually reflects a higher braking force.

### 3.4. Cluster Analysis

Cluster validation statistics indicated acceptable internal consistency in both analyses (cophenetic correlation = X.XX; average silhouette width = X.XX). In both models, the agglomeration schedule showed a marked increase in fusion coefficients at the transition from three to two clusters, supporting a two-cluster solution. The time 505 and time 303 variables consistently grouped with the same set of performance-related variables, indicating similar functional roles. As the analyses were exploratory, the identified clusters should be interpreted cautiously.

**Figure 2:**
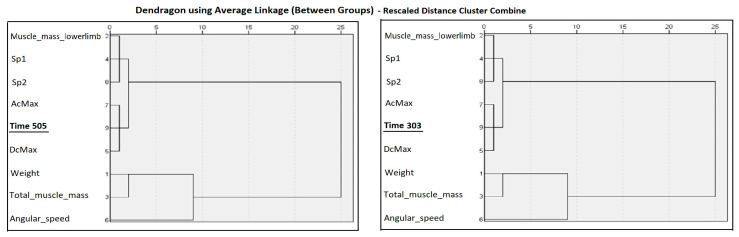
Hierarchical cluster structure of the studied variables in COD speed test and somatic variables—time 505/303.

The hierarchical cluster analysis (Figure 2) shows two broad branches in the dendrogram, separating (1) variables related to running performance and COD kinetics (Sp1, Sp2, AcMax, Time_303, and DcMax) from (2) anthropometric and muscle-mass indicators (Weight, total muscle mass). This visual structure suggests two high-level performance domains: a motor–kinetic component and an anthropometric component.

To evaluate the robustness of these groupings, we performed a silhouette analysis for 2–8 cluster solutions. The silhouette values increased steadily with the number of clusters (303 ms: 0.086 → 0.858; 505 ms: 0.086 → 0.871), indicating that finer-grained solutions provide better separation among variables. The highest silhouette values were obtained for the 8-cluster solution at both time points (303 ms: 0.858; 505 ms: 0.871), suggesting that the underlying structure of the data is more detailed than the two broad branches visible in the dendrogram.

The cophenetic correlations (303 ms: 0.733; 505 ms: 0.742) indicate good agreement between the dendrogram and the original proximity matrices, confirming that the hierarchical clustering reliably represents the similarity structure among variables.

### 3.5. General Linear Models for COD Time

The linear model fitted to the 505 ms run time was significant (F = 3.91, *p* = 0.006) and explained more than half of the variance (R^2^ = 0.533; adjusted R^2^ = XX). Among the predictor variables, only DcMax (maximum braking) showed a significant effect (*p* = 0.003), with a large effect size (partial η^2^ = 0.308). Effect sizes were interpreted according to Cohen’s benchmarks (small = 0.01, medium = 0.06, large = 0.14). Multicollinearity diagnostics indicated acceptable levels of collinearity (all VIF < 6; tolerance > 0.17; Table 5), and standard regression diagnostics (residual–fitted plots, Q–Q plots, Shapiro–Wilk tests) showed no major violations of assumptions.

**Figure 3:**
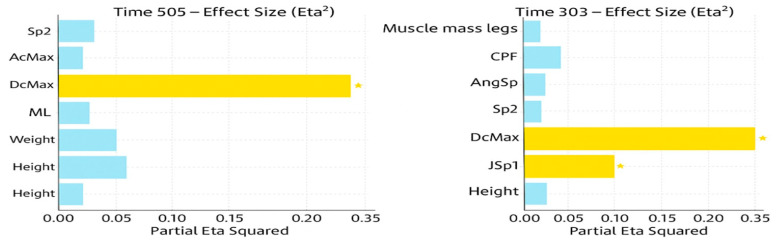
Illustration of DcMax as a primary predictor of COD performance.

The model for the 303 ms time point also reached significance (F = 2.34, *p* = 0.050), with moderate explanatory power (R^2^ = 0.490; adjusted R^2^ = XX). As in the 505 ms model, DcMax was the strongest predictor (*p* = 0.003, partial η^2^ = 0.344, large effect, Figure 3), and JSp1 (maximum speed before turning) also showed a significant contribution (*p* = 0.049, partial η^2^ = 0.129). Multicollinearity remained within acceptable limits (all VIF < 6.4; tolerance > 0.15; Table 6), and regression diagnostics again indicated no major assumption violations. These values correspond to the ones reported at the end of the tables.

### 3.6. Relationship Between DcMax and CMJ Parameters

Based on the correlations between CMJ parameters and maximum braking value (DcMax), only the impulse parameter (r = 0.386) showed a significant relationship with braking capacity. The relationship is positive, i.e., the higher the braking speed, the greater the impulse (Table 7).

## 4. Discussion

The primary aim of this study was to identify the mechanical, neuromuscular, and morphological determinants that most strongly influence change-of-direction (COD) performance in elite youth basketball players. By integrating DEXA-derived body composition, force-plate data, and high-frequency LPS tracking, we examined braking capacity, trunk rotational speed, centripetal force (CPF), and acceleration indices as potential predictors of COD performance. The findings revealed a clear and consistent pattern: maximal deceleration capacity (DcMax) was the strongest predictor of the 505 (303) COD test, whereas body composition variables and CMJ-derived performance measures played only secondary or negligible roles.

The central role of deceleration capacity aligns closely with the contemporary biomechanical literature. Previous studies have demonstrated that effective COD performance is primarily constrained by the quality of the braking step preceding the direction change. Dos’Santos [22] identified large eccentric braking forces and shorter ground-contact times as key differentiating factors between faster and slower performers. Harper [23] further described horizontal deceleration as a critical element in multidirectional sports, governed by neuromuscular coordination, rapid force development, and excentric strength. More recent conceptual frameworks [23,24] propose that braking should be considered an independent physical quality, rather than a secondary component of sprinting. Our predictive models reinforce this standpoint: DcMax accounted for the largest proportion of variance in COD time and remained the only consistent predictor across both tasks, supporting the view that “brake hard, turn fast” reflects a fundamental mechanical principle of direction-change performance.

In contrast, body composition demonstrated no significant relationship with COD outcomes. Although DEXA-based morphological traits are often associated with general physical performance such as sprinting or jumping [25,26,27], the present findings align with recent work indicating that anthropometric variables exert only indirect influence on COD performance. Bustamante-Garrido [28] and Singh [29] emphasize that COD ability arises from a multifactorial interaction among braking mechanics, neuromuscular control, approach speed, and technical execution, whereas anthropometric traits serve as structural characteristics with limited predictive utility. Our cluster analysis strongly supports this interpretation, revealing that anthropometric variables form a separate, independent cluster from functional COD-related mechanical variables. Thus, while body composition is important for global physical status, it is not a primary determinant of rapid direction-change ability in high-performance youth basketball.

The relationship between jump performance (CMJ) and COD ability yielded further insight. Several earlier studies reported moderate-to-strong correlations between CMJ performance and COD abilities [6,30,31]. However, these results frequently emerge in COD tests that emphasize re-acceleration or linear speed rather than braking. In the present study, CMJ-related variables displayed weak and non-significant relationships with both DcMax and COD time, suggesting that vertical power is not a meaningful predictor of braking-focused COD tasks. This observation is consistent with reports demonstrating that CMJ and COD correlations are task-specific and often weak when the deceleration phase dominates the movement demand [32]. Taken together, these findings underline that vertical jump performance, while reflective of global explosive capacity, does not capture the eccentric and mechanical demands of high-intensity braking actions.

An additional finding was the absence of right–left asymmetry in COD performance and associated mechanical variables. Although numerous studies have shown that inter-limb asymmetries exceeding 10–15% may impair performance or elevate injury risk [33,34], asymmetries are not universal and tend to be minimal in well-trained, technically proficient athletes. Our results indicate that no statistically detectable bilateral asymmetry was observed in this cohort. This suggests that, within the sensitivity of the applied tests, players performed COD tasks similarly on both sides. However, these findings should not be interpreted as evidence of symmetry resulting from long-term training adaptations, as this cannot be inferred without longitudinal data

The multidimensional nature of COD performance was evident in the cluster structure of the measured variables. Anthropometric characteristics formed a distinct cluster, while braking, approach speed, and reacceleration variables comprised a functional COD-related cluster. This structural separation is consistent with systematic reviews highlighting that COD ability is composed of interrelated but biomechanically distinct subcomponents [29,35]. The present findings therefore provide empirical support for the notion that COD performance is not governed by a single physical trait but emerges from the coordinated interaction of braking mechanics, technical execution, and neuromuscular control.

Previous statistics and studies confirmed the increase in the amount, speed and role of COD in practically most team and individual ball sports. In basketball, movement elements such as the euro step, sidestep, and step back are becoming increasingly dominant; all of these are based on the turning step and are preceded by a significant amount of braking. It is already well-known and proven by measurements in elite sports [36,37,38,39] that jump-based plyometric training programmes have a positive effect on the performance in this kind of agility-type tasks, but we would like to obtain more specific, detailed information. We examined our deceleration data and the CMJ indicators in a separate correlation matrix, where we found a clear relationship between the maximum deceleration of the 505 test and the CMJ Impulse data. Impulse is the sudden “shock” occurring at the moment of eccentric and concentric change, which in our CMJ study refers to the moment between the descent and the ascent phase. This is practically an SSC cycle, where it seems logical that the more dynamic the descent, the more dynamic the ascent. However, we can only add great dynamics to the lowering phase if we can apply a sufficient amount of braking before the eccentric/concentric change; otherwise, we cannot transfer the energy to the take-off (concentric phase). Based on the present findings, high braking demands appear to be closely associated with COD performance, which suggests a potential practical implication: training methods that target braking capacity may be relevant for improving COD ability. However, this interpretation should be viewed as a practical consideration rather than a demonstrated training effect, as no longitudinal or intervention data were collected in this study.

Collectively, this study advances current understanding by demonstrating that braking efficiency—not body composition or vertical power—is the key mechanical bottleneck of COD performance in elite youth basketball players. The use of LPS-derived braking metrics offers novel insights and may enhance the ability to assess or monitor COD-related capacities in highly specific, sport-relevant contexts.

### Limitations

The relatively small sample size of elite U18 male basketball players limited statistical power and increased the risk of model overfitting, particularly in the multivariable analyses. Although variance inflation factors did not indicate problematic multicollinearity, the possibility of shared variance among mechanical variables could not be fully excluded. Biological maturation of basketball players was not included as a covariate, which might have influenced braking capacity, strength, and body-composition characteristics in late-adolescent athletes. Although all tests demonstrated acceptable within-session reliability, we did not report between-session reliability for the mechanical variables derived from the local positioning system. The findings could not be generalized beyond elite Hungarian U18 males; different age groups, competitive levels or female athletes may show different mechanical determinants of change-of-direction performance. It can be said that the level of COD plays an important role primarily in one-on-one situations, both on the offensive and defensive side, and education and development of skills relating to COD are effective tools in the training of athletes.

In addition, the number of predictors relative to the sample size increases the risk of model instability, and the cross-sectional design prevents any causal inference regarding the relationships observed. The study also involved multiple statistical tests, which may increase the likelihood of Type I error despite the consistent patterns observed. These factors should be considered when interpreting the independent contribution of each variable to COD performance.

Furthermore, although the local positioning system (LPS) enabled high-frequency tracking of deceleration, centripetal force and trunk-rotation metrics, these variables still represent indirect estimates of underlying biomechanical processes. LPS cannot capture joint-level kinetics or muscle-specific eccentric actions, which limits the precision with which braking impulse and technique can be quantified.

Finally, although body-composition variables did not show independent associations with COD performance in our models, this finding should be interpreted cautiously. The combination of modest sample size and shared variance among anthropometric measures may have reduced their independent predictive power in the general linear models. Therefore, our results indicate only that body composition showed no independent association with COD performance after controlling for braking-related variables, rather than suggesting that anthropometry is unimportant in COD ability.

## 5. Conclusions

This study identified maximal deceleration capacity (DcMax) as the variable most strongly associated with change-of-direction (COD) performance in elite youth basketball players. DcMax consistently emerged as the strongest predictor of COD times in both the 505 and 303 tasks, whereas DEXA-derived morphological variables and CMJ-based explosive performance measures showed no independent associations after accounting for braking-related metrics. No statistically detectable right–left differences were observed in this cohort.

These findings suggest that COD performance in this cohort is most closely associated with braking efficiency, rather than with body-composition characteristics or vertical jump performance. While braking capacity appears to play a prominent role in COD tasks, these associations should not be interpreted as causal due to the cross-sectional design and the potential influence of shared variance among predictors. The cluster analysis further indicates that COD ability reflects the interaction of braking mechanics, neuromuscular control, and technical execution, rather than being primarily explained by morphological characteristics.

From a practical standpoint, these results highlight braking-related qualities as potentially relevant targets for training, although intervention studies are needed to confirm their effectiveness. Eccentric strength development, braking-specific plyometric exercises, and technical refinement of deceleration mechanics may hold promise, but their direct transfer to high-speed COD performance requires further investigation.

Overall, this study provides evidence that braking-related variables are the strongest predictors of COD performance in elite youth basketball players, while emphasizing the need for longitudinal and experimental research to establish causal relationships and training effects.

## Figures and Tables

**Table 1:** Descriptive statistics of anthropometric and body composition variables.

	N	Shapiro–Wilk Test (p)	Range of Stat	Mean	Median	Minimum	Maximum	SD
Age (years)	32	0.597	3.17	17.32	17.63	16.04	19.21	0.9
Height at Exam (cm)	32	0.320	15.3	194.84	193.7	187.1	202.4	4.54
Weight at Exam (kg)	32	0.053	27.8	89.13	87.2	77.4	105.2	9.43
BMI (kg/m^2^)	32	0.472	5.8	23.43	23.8	20.9	26.7	1.75
Total Body Fat (kg)	32	0.312	14.27	15.23	15.62	8.48	22.75	4.59
Total Body Muscle (kg)	32	0.521	21.06	69.98	70.87	60.34	81.4	6.7
Lower Limb Muscle(kg)	32	0.270	7.07	24.52	24.46	21.46	28.53	2.26

**Table 2:** Descriptive statistics of functional performance and mechanical variables.

	N	Shapiro–Wilk Test (p)	Range of Stat	Mean	Median	Minimum	Maximum	SD	Std. Error of Mean
Time_505	32	0.558	0.31	2.36	2.38	2.18	2.49	0.09	0.02
Time_303	32	0.969	0.34	1.84	1.85	1.65	1.99	0.08	0.01
Sp1	32	0.768	3.58	22.05	21.95	20.06	23.64	0.81	0.14
DcMax	32	0.600	1.98	−7.26	−7.22	−8.3	−6.32	0.52	0.09
AngSp	32	0.107	68.1	125.54	127.83	97.1	165.2	17.8	3.15
CPF	32	0.130	449.21	590.6	583.24	407.85	857.06	102.22	18.07
AcMax	32	0.120	1.35	6.28	6.34	5.55	6.9	0.38	0.07
Sp2	32	0.561	4.49	18.33	18.27	16	20.49	1.14	0.2
H	32	0.121	0.2	0.45	0.48	0.34	0.54	0.06	0.01
D	32	0.21	0.15	0.36	0.36	0.28	0.43	0.04	0.01
Speed	32	0.213	0.76	2.92	3	2.47	3.23	0.19	0.03
Im	32	0.242	184	484.69	487.5	395	579	50.15	8.86
PMR	32	0.463	31.3	62.99	63.9	47.1	78.4	7.67	1.36
PAR	32	0.136	16.6	32.04	32.05	23.9	40.5	4.68	0.83
LR	32	0.061	73	68.06	70.5	24	97	20.66	3.65
FB	32	0.244	64	185.44	185	148	212	16.41	2.9

Time_505—505 test running time (s); Time_303—505 test shorter etap measured running time (s); Sp1—sprint before turning maximum speed (m/s); Sp2—sprint after turning maximum speed (m/s); DcMax—maximum deceleration before turning (m·s^−2^); AngSp—angular speed (°/s); CPF—centripetal force (Newton); AcMax—maximum acceleration after turning (m·s^−2^); H—CMJ height (m); D—move down before jump (m); Speed—speed up way during jump (m/s); Im—impact during change from excentric to concentric phase (N·s); PMR—Power Max Relative, maximum power load from player during jump (W/Kg); PAR—Power Average Relative, average power load from player during jump (W/Kg); LR—lateral movement of centre of body mass before leave the force plate during jump (mm); FB—lineal movement of centre of body mass before leave the force plate during jump (mm).

**Table 3:** Significance levels of paired samples *t*-test—between right and left turns of individuals.

Tasks		Shapiro–		Paired Differences						df	Sig. (2-Tailed)	Cohen’s d
		Wilk Test (p)		Mean	Std. Deviation	Std. Error Mean	Interval of the		t			
		R	L				Lower	Upper				
Pair 1	Right505-Left505	0.428	0.354	−0.007	0.102	0.018	−0.044	0.030	−0.373	31	0.711	0.07
Pair 2	Right303-Left303	0.690	0.524	0.003	0.104	0.018	−0.034	0.040	0.171	31	0.866	0.03
Pair 3	RightSp1-LeftSp1	0.719	0.712	−0.055	0.449	0.079	−0.217	0.107	−0.692	31	0.494	0.12
Pair 4	RightDxMax-LeftDcMax	0.342	0.393	0.027	0.544	0.096	−0.170	0.222	0.273	31	0.787	0.05
Pair 5	RightAngSp-LeftAngSp	0.215	0.493	−1.231	27.168	4.803	−11.026	8.564	−0.256	31	0.799	0.05
Pair 6	RightCPF-LeftCPF	0.070	0.056	−18.092	135.742	23.996	−67.032	30.848	−0.754	31	0.457	0.13
Pair 7	RightAcMax-LeftAcMax	0.211	0.286	−0.016	0.356	0.063	−0.144	0.113	−0.249	31	0.805	0.04
Pair 8	RightSp2-LeftSp2	0.672	0.910	0.173	0.843	0.149	−0.131	0.477	1.161	31	0.254	0.21

**Table 4:** Pearson correlation coefficients between the variables (multiple comparison-adjusted significance using Benjamini–Hochberg FDR; bold values indicate q < 0.05).

	Weight	Leg Muscle	Body Muscle	Time 505	Time 303	Sp-1	DcMax	AngSp	CPF	AcMax	Sp-2
Height-r	**0.767**	**0.595**	**0.533**	0.427	0.366	**−0.441**	**0.378**	−0.313	0.067	**−0.633**	**−0.270**
q	<0.001	<0.001	0.045	0.628	0.748	0.033	0.051	0.135	0.748	0.002	0.047
Weight-r	-	**0.845**	**0.850**	0.361	0.246	**−0.482**	**0.358**	−0.185	**0.376**	**−0.618**	−0.164
q		<0.001	<0.001	0.340	0.289	0.017	0.048	0.468	0.035	0.002	0.371
Leg Muscle-r		-	**0.956**	0.232	0.106	**−0.528**	**0.407**	−0.225	**0.431**	**−0.585**	−0.095
q			<0.001	0.748	0.540	0.009	0.050	0.341	0.041	0.012	0.606
Body Muscle-r			-	0.195	0.060	−0.506	0.369	−0.157	**0.407**	**−0.497**	−0.002
q				0.540	0.340	0.135	0.628	0.412	0.032	0.017	0.786
Time 505-r				-	**0.958**	−0.445	**0.644**	−0.238	−0.014	−0.448	**−0.309**
q					<0.001	0.628	0.033	0.628	0.748	0.468	0.015
Time 303-r					-	−0.350	**0.538**	−0.189	−0.065	−0.345	−0.191
q						0.748	0.049	0.776	0.412	0.412	0.890
Sp-1-r						-	**−0.718**	0.151	−0.084	**0.553**	**0.509**
q							0.002	0.628	0.748	0.035	0.003
DcMax-r							-	**−0.327**	0.167	−0.636	−0.291
q								0.045	0.504	0.628	0.107
AngSp-r								-	0.068	0.378	**0.319**
q									0.748	0.468	0.033
CPF-r									-	**0.585**	0.134
q										0.040	0.466
AcMax-r										-	**0.454**
q											0.023

**Table 5:** General linear model results—Time 505 (VIF: variance inflation factor, *: multicollinearity diagnostics, df = 24; bold values indicate *p* < 0.05).

Source	Type III Sum of Squares	df	Mean Square	F	Sig.	Partial Eta Squared	Tolerance *	VIF *
Corrected Model	0.122	7	0.017	3.912	**0.006**	0.533		
Intercept	0.018	1	0.018	4.055	0.055	0.145		
Height	0.002	1	0.002	0.513	0.481	0.021	0.352	2.841
Weight	0.007	1	0.007	1.686	0.207	0.066	0.174	5.741
ML	0.006	1	0.006	1.453	0.240	0.057	0.233	4.301
JSp1	0.003	1	0.003	0.655	0.426	0.027	0.291	3.435
DcMax	0.047	1	0.047	10.662	**0.003**	0.308	0.337	2.964
AcMax	0.003	1	0.003	0.589	0.450	0.024	0.309	3.241
Sp2	0.004	1	0.004	0.994	0.329	0.040	0.525	1.904
Error	0.107	24	0.004					
Total	178.899	34						
Corrected Total	0.228	33						
R Squared = 0.533								

**Table 6:** General linear model results—Time 303 (VIF: variance inflation factor, *: multicollinearity diagnostics, df = 22; bold values indicate *p* < 0.05).

Source	Type III Sum of Squares	df	Mean Square	F	Sig.	Partial Eta Squared	Tolerance *	VIF *
Corrected Model	0.106	9	0.012	2.344	0.050	0.490		
Intercept	0.002	1	0.002	0.352	0.559	0.016		
Height	0.003	1	0.003	0.643	0.431	0.028	0.313	3.198
Weight	0.006	1	0.006	1.238	0.278	0.053	0.156	6.398
JSp1	0.016	1	0.016	3.266	**0.049**	0.129	0.232	4.304
DcMax	0.058	1	0.058	11.544	**0.003**	0.344	0.270	3.704
AcMax	0.005	1	0.005	1.016	0.324	0.044	0.288	3.470
Sp2	0.005	1	0.005	0.986	0.331	0.043	0.455	2.199
AngSp	0.002	1	0.002	0.456	0.507	0.020	0.659	1.518
CPF	0.007	1	0.007	1.296	0.267	0.056	0.608	1.644
Muscle mass legs	0.002	1	0.002	0.415	0.526	0.019	0.201	4.983
Error	0.11	22	0.005					
Total	108.887	34						
Corrected Total	0.216	31						
R Squared = 0.490								

**Table 7:** Pearson correlation coefficients of CMJ parameters with DcMax (the bold coefficient represents significant correlation, bold value indicates *p* < 0.05).

CMJ Parameters		r
H	height of the CMJ (m)	−0.277
D	lowering of the CoG (m)	−0.124
Sp	the velocity of take-off (m/s)	−0.291
Im	impulse (N × s)	**0.386**
PMR	relative max power (Watt/kg)	−0.302
PAR	relative mean power (Watt/kg)	−0.296
LR	the CoG moving lateral (mm)	−0.219
FB	the CoG moving linear (mm)	0.058

## Data Availability

The datasets generated and analyzed during the current study are not publicly available due to personal data of the participants, but are available from the Corresponding Author upon reasonable request.

## Abbreviations

Age	chronological age (years)
Height at Exam	body height (cm)
Weight at Exam	body weight (kg)
BMI	body mass index (kg·m^−2^)
Total Body Fat	total body fat (kg)
Total Body Muscle	total body muscle mass (kg)
Lower Limb Muscle	lower limb muscle mass (kg)
505	505 Agility test 5-5 m distance time result (s)
303	505 Agility test shorter 3,5-3,5 m distance time result (s)
Sp1	sprint before turning, maximum speed (m·s^−1^)
DcMax	maximum deceleration (m·s^−2^)
AngSp	angular speed of trunk rotation during change of direction (°·s^−1^)
CPF	centripetal force during change of direction (N)
AcMax	maximum acceleration after turning (m·s^−2^)
Sp2	sprint after turning, maximum speed (m·s^−1^)
H	CMJ height (m)
D	downward movement before jump (m)
Speed	maximum upward speed during jump (m·s^−1^)
Im	impulse at the eccentric–concentric transition (N·s)
PMR	relative maximal power during jump (W·kg^−1^)
PAR	relative average power during jump (W·kg^−1^)
LR	lateral displacement of the centre of gravity before take-off (mm)
FB	anteroposterior displacement of the centre of gravity before take-off (mm)
